# Breast cancer survival prediction using an automated mitosis detection pipeline

**DOI:** 10.1002/2056-4538.70008

**Published:** 2024-10-28

**Authors:** Nikolas Stathonikos, Marc Aubreville, Sjoerd de Vries, Frauke Wilm, Christof A Bertram, Mitko Veta, Paul J van Diest

**Affiliations:** ^1^ Pathology University Medical Centre Utrecht Utrecht The Netherlands; ^2^ Technische Hochschule Ingolstadt Ingolstadt Germany; ^3^ Digital Health University Medical Centre Utrecht Utrecht The Netherlands; ^4^ Information and Computing Sciences Utrecht University Utrecht The Netherlands; ^5^ Pattern Recognition Lab Friedrich‐Alexander‐Universität (FAU) Erlangen‐Nürnberg Erlangen Germany; ^6^ Institute of Pathology University of Veterinary Medicine Vienna Vienna Austria; ^7^ Medical Image Analysis Group TU Eindhoven Eindhoven The Netherlands

**Keywords:** artificial intelligence, machine learning, histopathology, prognosis, mitosis, outcome, deep learning

## Abstract

Mitotic count (MC) is the most common measure to assess tumor proliferation in breast cancer patients and is highly predictive of patient outcomes. It is, however, subject to inter‐ and intraobserver variation and reproducibility challenges that may hamper its clinical utility. In past studies, artificial intelligence (AI)‐supported MC has been shown to correlate well with traditional MC on glass slides. Considering the potential of AI to improve reproducibility of MC between pathologists, we undertook the next validation step by evaluating the prognostic value of a fully automatic method to detect and count mitoses on whole slide images using a deep learning model. The model was developed in the context of the Mitosis Domain Generalization Challenge 2021 (MIDOG21) grand challenge and was expanded by a novel automatic area selector method to find the optimal mitotic hotspot and calculate the MC per 2 mm^2^. We employed this method on a breast cancer cohort with long‐term follow‐up from the University Medical Centre Utrecht (*N* = 912) and compared predictive values for overall survival of AI‐based MC and light‐microscopic MC, previously assessed during routine diagnostics. The MIDOG21 model was prognostically comparable to the original MC from the pathology report in uni‐ and multivariate survival analysis. In conclusion, a fully automated MC AI algorithm was validated in a large cohort of breast cancer with regard to retained prognostic value compared with traditional light‐microscopic MC.

## Introduction

The annual global incidence of breast cancer (BC) exceeds 2 million cases, making it the most commonly diagnosed cancer worldwide [1]. Female BC currently ranks fifth in terms of mortality on a global scale, and its incidence continues to rise. Nonetheless, when detected in its early stages, BC can have a favorable prognosis. The main determinants of BC prognosis are typically tumor size, lymph node status, and histopathological grade [2, 3]. Of these, histopathological grade is typically evaluated using the Nottingham modification of the Bloom–Richardson (BR) grade [4, 5]. The BR grading system involves assessing three key features: tubule formation, nuclear pleomorphism, and mitotic count (MC). Each feature is assigned a score ranging from 1 to 3. Score sums in the range of 3–5 classify the cancer as grade 1, score sums of 6–7 as grade 2, and score sums of 8–9 as grade 3 BC. Grade 1 BCs generally exhibit significantly better survival rates than grade 2 or 3 cancers [2, 3, 4]. Furthermore, the histopathological grade has been shown to influence treatment decisions in up to a third of cases [2]. Among the components of BR grade, MC, as a marker of tumor proliferation, is the most prominent and a high MC is associated with poor prognosis [6, 7, 8, 9]. However, assessing MC is an error‐prone process that requires strict protocols to be highly reproducible [10]. According to guidelines [11], mitoses should be counted at ×40 objective magnification in the most mitotically active part of the tumor in an area of approximately 2 mm^2^ where most mitotic cells are found, the so‐called mitotic hotspot. Next, specific cut‐offs are applied to calculate the MC, which is categorized as 1, 2, or 3. Still, various studies have reported only moderate reproducibility for the BR grading system [12, 13, 14]. A recent study identified significant inter‐ and intralaboratory variations in BR grade among more than 33,000 patients [12]. Given these variations and the critical role of MC in BC prognosis, achieving higher reproducibility is required for optimal clinical applicability of MC and BR grade.

In recent years there have been several studies [15, 16, 17] as well as grand challenges [18, 19, 20] around the detection of mitotic cells in invasive BC using machine learning algorithms, with excellent results reaching human observer performance [19, 20]. In a previous study, we showed that an artificial intelligence (AI)‐based mitoses detector achieved similar diagnostic outcomes of MC assessment [15]. AI algorithms have great potential to improve reproducibility [16, 21] and efficacy of MC since they can analyze multiple whole slide images (WSIs) and help the pathologist to quickly find the optimal hotspot saving considerable amounts of time. However, AI assisted MC is not yet widely implemented in clinical practice, which makes it difficult to assess its added value on a broader scale. This lack of implementation can be attributed to technical difficulties such as not having a fully digital workflow, lack of integration into image management systems, necessity of specialized IT infrastructure and personnel, but especially lack of prognostic validation of available AI‐based MC models [22, 23].

For the present study, we therefore aimed to investigate if an automated AI‐based method to assess MC on hematoxylin and eosin (H&E)‐stained WSIs of BC is prognostically noninferior to traditional MC on glass slides using existing internationally recognized diagnostic criteria.

## Materials and methods

### Study cohort

We collected a large digital pathology and clinical BC dataset from a single source (University Medical Centre [UMC] Utrecht) with follow‐up for up to 10 years from patients treated in our hospital from 2000 until 2013 (*n* = 2,230). We excluded WSIs not scanned at ×40 magnification or that had issues with scan quality (significant amount of out‐of‐focus regions, tissue folds, and tissue tears), cases above the age of 80 years, or where MC was missing in the original pathology reports, finally leaving 912 unique subjects (see Tables 1 and 2). Slides were scanned with a Hamamatsu XR NanoZoomer 2.0 at a resolution of 0.23 μm/pixel, using a ×40 objective lens. For every case, there was at least one slide where a pathologist roughly annotated the tumor outline to confine the processing area. Since light‐microscopic MC in the original pathology reports was reported as the number of mitoses per 2 mm^2^, we implemented MC by the AI models accordingly.

**Table 1:** Dataset overview – explanation of inclusion criteria for the study

	Included	Excluded
Total cases	2,230	0
Only ×40 scans	1,466	764
With mitotic activity index and survival data	1,316	150
Correctly labeled and of sufficient quality	1,237	79
Below 81 years old	1,005	232
Unique subjects	912	

**Table 2:** Breakdown of the type of cases included in the cohort

	*n*	%
Total subjects	912	100
Sex		
Male	6	0.7
Female	906	99.3
Age, years		
<50	309	33.9
≥50	603	66.1
Histopathological type		
Ductal	708	77.6
Lobular	74	8.1
Ductolobular	93	10.2
Unknown	37	4.1
Immunohistochemical subtype		
Luminal A	300	32.9
Luminal B	114	12.5
Triple‐negative	72	7.9
HER2‐driven	20	2.2
Unknown	406	44.5

### Mitosis detection pipeline

**Figure 1:**
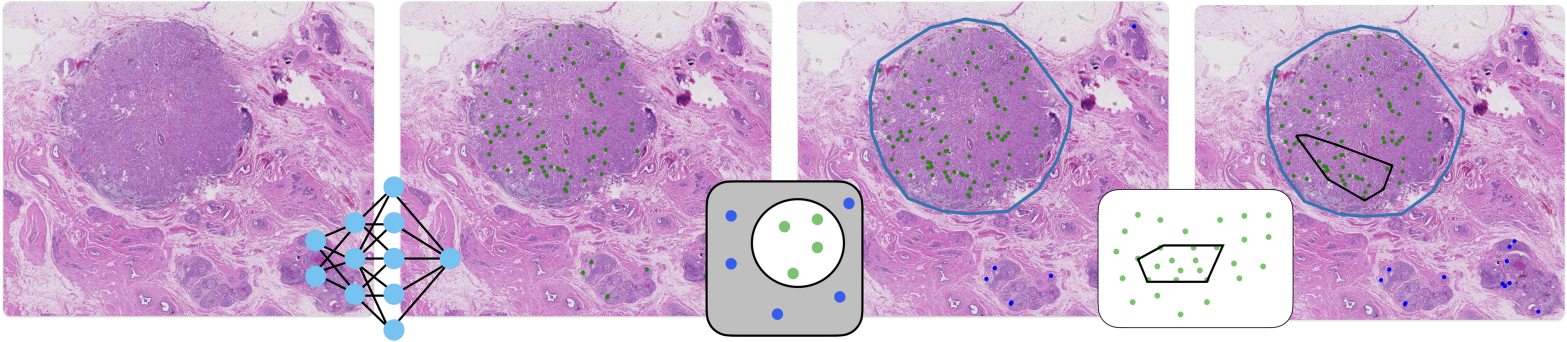
Pipeline of the detection model. The model first detects all mitotic objects in the image. The objects that are outside of the tumor annotation are excluded and the automatic area selector is run on the remaining objects to calculate the final MC.

To detect mitotic figures (MFs) in the H&E slides, we employed an AI model based on a convolutional neural network that scans a WSI for possible mitotic cells and assigns a probability to all identified candidates. Using a predetermined threshold, we classified all detections above a certain threshold as MFs that were subsequently used as input to calculate the MC. Using the tumor annotations supplied by the pathologists, we excluded all candidates outside of the tumor area. Using the remaining MFs we applied an area detection algorithm (see below) to determine the 2 mm^2^ area of the mitotic hotspot according to the modified BR scoring system (see Figure 1), and the number of MFs herein was used as the final MC detected per WSI. The AI model was developed using the dataset [24] from the Mitosis Domain Generalization Challenge 2021 (MIDOG21), which focused on mitotic cell detection in the presence of scanner‐induced domain shifts [19, 25]. In the context of the challenge, a baseline model was provided by the organizers, trained on the official challenge training dataset. This model achieved great performance and ranked within the top 5 methods of the challenge [19] while achieving a higher *F*_1_‐score than the majority of the human experts on the challenge test set [19]. The MIDOG21 model was based on a the RetinaNet architecture [26] which was customized by adding a gradient reversal layer (GRL) and a domain classifier [25]. The domain classifier was trained in an adversarial fashion to classify the different WSI scanners available in the dataset, aiming to make the model applicable on all scanners. Using a GRL, the feature encoder was trained to extract domain‐invariant features. The network was trained on the grand challenge training dataset and evaluated on the preliminary test dataset. The threshold used was selected by maximizing the *F*_1_‐score on an internal validation subset of the MIDOG challenge training data, which corresponds to an *F*_1_‐score of 0.7369 at an operating point of 0.64 [25]. The model has been published and the code and weights are available online. (https://github.com/DeepMicroscopy/MIDOG_reference_docker).

### Automatic area computation

In order to determine the MCs, a pathologist must find the tumor area with the highest mitotic activity and count all mitotic cells at ×40 objective magnification up to an area of 2 mm^2^ [10]. To emulate this diagnostic step, a procedure to automatically select this area had to be developed. We used two different methods to determine the highest mitotic activity area, an overlapping window search algorithm and a geometric computation method for calculating optimal convex hulls.

### Overlapping window search

For the window search we employed a fixed area size rectangle of 2 mm^2^ with a 4:3 ratio of width over height. Using this rectangular search area we iterated over the MF detections with an overlap of a quarter of the width and height of the rectangle and counted all of the MFs that were found within. The rectangle with the most MF detections was selected as the MC for that slide.

### Convex hull geometric calculation

For the method employing the geometric calculation, we opted for a convex polygon as the shape of the area, as this was thought to be more natural and comparable to the area that might normally be inspected by a pathologist, rather than fixed geometric shapes such as a circle and triangle, which are more likely to include a part of the image where no tissue is present. The automatic area computation algorithm, which we named ‘bounded area maximum enclosing convex hull’ (BAME convex hull), is based on a gift wrapping algorithm from computational geometry [27]. A bound on the aspect ratio of the resulting polygon was desired to ensure the convex hull was not thinly stretched, i.e. unnatural, so the WSI was subdivided into square patches with an area of 4 mm^2^, with an overlap of 0.5 mm. This limited size causes any shape that spans the whole width to still have room for an average width of 0.5 mm that can be used to include more points, before the area constraint is violated. Furthermore, this patch‐based processing significantly sped up the computation, since the computational burden of finding an optimal solution sharply increases with the number of points. Nevertheless, finding an exact solution remains impractical for patches that contain a large number of points. To ensure that performance was acceptable for use in clinical practice, two algorithms were designed: An exact algorithm that was used on patches that contained 25 or fewer points and a heuristic one that was applied to patches with more points. The precise implementations of these algorithms can be found on github (https://github.com/sjoerd-de-vries/Area-Selector) and their workings are explained in both text and pseudocode in Supplementary materials and methods and Figure S1.

### Case‐level tumor proliferation scoring

When multiple slides were available for a case, the slide with the highest MC was selected by the model emulating the regular diagnostic workflow. For patients with multiple tumors, we selected again the tumor with the highest MC. The selected MC per case was then translated into the mitosis score of the BR grading system following the rules of the College of American Pathologists guidelines: a score of 1 for MC of 7 and below, score 2 for MC between 8 and 14 and score 3 for MC 15 and higher [11].

### Statistical analysis

Statistical analysis was performed using lifelines [28], scipy [29], scikit‐learn [30] on python 3.9. We compared the light‐microscopic MC from the original pathology reports to the AI‐based MC as continuous variables by Pearson correlation. The agreement between the three‐class BR MC scores was assessed by linearly weighted Cohen's kappa. For comparing overall survival prediction of the different MC variables, a 5‐year survival difference Kaplan–Meier estimator using a Klein *et al* [31] transformation was used. In addition, to assess the prognostic significance of MC scores in relation to other diagnostic parameters, we performed a multivariate survival analysis by Cox regression analysis.

## Results

### Correlation between original light‐microscopic MCs and automatic AI‐based counts

**Figure 2:**
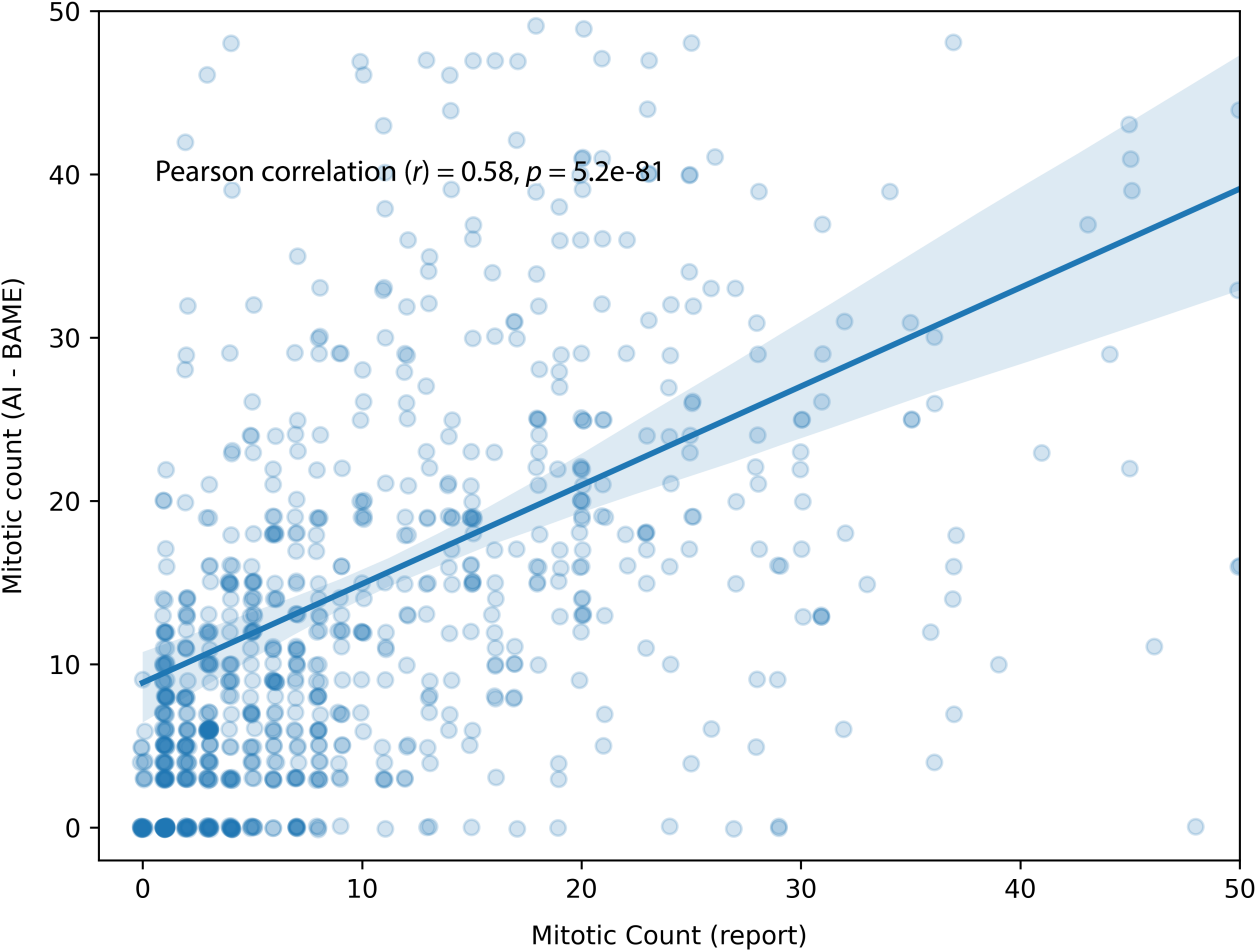
Regression between AI‐based MC and report.

AI‐based MC showed a strong correlation with the light‐microscopic MC from the original report (Pearson *r* = 0.58, *p <* 0.00001), as shown in Figure 2. Agreement between AI‐based BR mitotic score and light‐microscopic BR mitotic score was similar (*κ* = 0.5). For the AI‐based MC using the overlapping window search, the results were also similar (Pearson *r* = 0.57, *p <* 0.00001) and Cohen's kappa (*κ* = 0.52).

### Comparison of prognostic value

**Figure 3:**
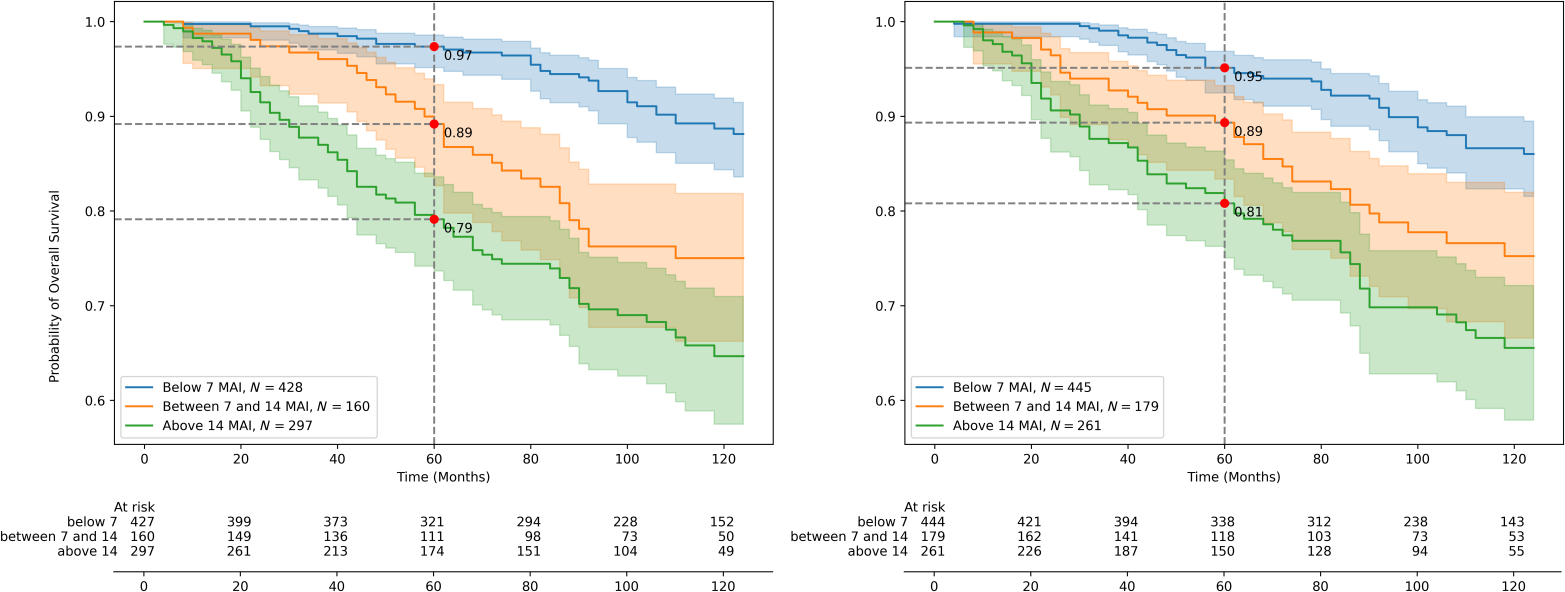
Kaplan–Meier estimates (overall survival) for original Bloom–Richardson (BR) mitotic score (left) versus automatic AI‐based (BAME method) mitotic scores (right) showing comparable survival estimates for the three BR mitotic score classes.

**Figure 4:**
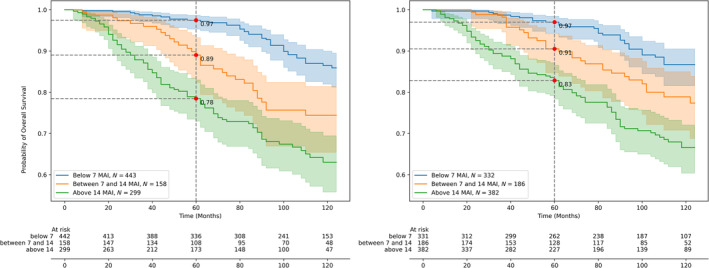
Kaplan–Meier estimates (overall survival) for original Bloom–Richardson (BR) mitotic score (left) versus automatic AI‐based (window search method) mitotic scores (right) showing comparable survival estimates for the three BR mitotic score classes.

Figures 3 and 4 show overall survival curves for light‐microscopic and AI‐based BR mitotic scores. For all methods, the curves for the three scores diverge significantly with *χ* ^2^ = 12.55 (*p <* 0.001) and *χ* ^2^ = 5.70 (*p <* 0.01) for light‐microscopic mitotic score groups (1, 2) and (2, 3), respectively; and, for the AI‐based methods, *χ* ^2^ = 7.22 (*p <* 0.01) and *χ* ^2^ = 4.59 (*p =* 0.03) for the AI‐based BAME method and *χ* ^2^ = 5.51 (*p =* 0.018) and *χ* ^2^ = 4.58 (*p =* 0.03) for the AI‐based window search method, for groups (1, 2) and (2, 3), respectively. In multivariate Cox regression analysis we included age, tumor size, lymph node status (if lymph nodes were positive for tumor metastasis), and BR mitosis scores as covariates. Both light‐microscopic and both AI‐based BR mitotic scores retained additional prognostic value (see Tables 3 and 4).

**Table 3:** Multivariate Cox regression statistics on report and AI‐based BAME and window search mitotic count

	Report		AI (BAME)		AI (window search)	
	Hazard ratio (95% CI)	*p*	Hazard ratio (95% CI)	*p*	Hazard ratio (95% CI)	*p*
Mitosis count	0.99 (0.97–1.00)	0.08	1.00 (0.99–1.01)	0.67	1.00 (0.99–1.02)	0.49
Age	1.02 (1.01–1.04)	<0.005	1.02 (1.01–1.04)	<0.005	1.02 (1.01–1.03)	0.01
Lymph node status	1.85 (1.32–2.58)	<0.005	1.76 (1.26–2.46)	<0.005	1.86 (1.32–2.64)	<0.005
Tumor size	1.26 (1.18–1.35)	<0.005	1.23 (1.15–1.31)	<0.005	1.25 (1.17–1.33)	<0.005
Mitotic score						
Score 1 (0–7)	1 (Ref)		1 (Ref)		1 (Ref)	
Score 2 (8–14)	2.14 (1.38–3.31)	<0.005	1.41 (0.86–2.30)	0.17	1.70 (1.10–2.63)	0.02
Score 3 (15 and higher)	4.26 (2.62–6.95)	<0.005	2.35 (1.46–3.79)	<0.005	2.18 (1.30–3.65)	<0.005

**Table 4:** Univariate Cox regression statistics on report and AI‐based BAME and window search mitotic count

	Report		AI (BAME)		AI (window search)	
	Hazard ratio (95% CI)	*p*	Hazard ratio (95% CI)	*p*	Hazard ratio (95% CI)	*p*
Mitotic score						
Score 1 (0–7)	1 (Ref)		1 (Ref)		1 (Ref)	
Score 2 (8–14)	2.09 (1.37–3.19)	<0.005	1.64 (1.02–2.63)	<0.005	2.19 (1.44–3.31)	<0.005
Score 3 (15 and higher)	3.26 (2.31–4.60)	<0.005	3.13 (2.15–4.55)	<0.005	3.07 (2.15–4.40)	<0.005

### Influence of pathologist supervision on AI model output

In 30 cases, an experienced pathologist (PJvD) reviewed all individual objects found by the model in the tumor area, reclassified the output, re‐ran the automatic area selector, and performed a final review of the objects in the selected area. In this subgroup, the Pearson correlation between pathologist‐corrected AI‐based BR mitotic score and original report MC was *r* = 0.505 (*p* = 0.0044) while the correlation between uncorrected AI model output (no intervention by pathologist) and BR mitotic score from the original report was *r* = 0.40 (*p* = 0.028) with kappa being 0.43 and 0.21 respectively.

## Discussion

Grading is still a very effective way [2, 3, 4] to accurately offer a prognosis for a BC patient. Mitosis counting is the most important constituent of grading and is also directly correlated to patient outcome [7]. However, modest interobserver agreement is a considerable drawback of MC, which will likely improve with AI, but retained prognostic value must be demonstrated to avoid the ‘precise but not accurate’ caveat. In our study, we compared the prognostic value of light‐microscopic MC as reported in the original pathological report, with MC by an automated AI algorithm. In both uni‐ and multivariate survival analysis, AI‐based MC had comparable prognostic value to light‐microscopic MC. When comparing the two AI methods, that differ only on how the MC is derived, the window search overlap method performed slightly better than the BAME convex hull method. The hazard ratios for both groups (1, 2) and (2, 3) for the window search overlap were independent predictors of prognosis, whereas for the BAME method, the *p* value for group (1, 2) was above 0.05.

From this we see that the AI model itself (regardless of the MC method) performs on par diagnostically with the light‐microscopic method without having to revise or devise new diagnostic criteria, simply following existing guidelines.

Our AI mitoses detection model was derived from the MIDOG21 grand challenge that intended to foster an environment for developing the most appropriate AI methods to detect mitotic cells across domains be that scanners, tissue types, or even species [20]. The developed models were made publicly available, first to the grand challenge participants and then to the greater public (https://github.com/DeepMicroscopy/MIDOG_reference_docker). When comparing the method developed by Wilm *et al* [25] for MIDOG21, it performed as well as some of the top submissions using an established methodology and network structure [26]. It is opensource and the model weights are available, so it can be used by others to further validate and perhaps improve upon using other datasets. We have fully integrated the model within our picture archiving and communication system (PACS) and have been using it in clinical practice at the UMC Utrecht in order to properly evaluate and certify it internally in compliance with our quality system. A limitation of this model is that it was only trained with hotspot regions of interests and not whole tumor or entire WSI‐labeled datasets; this makes it perhaps weaker at recognizing some artifacts and generating false positive detections that most often occur outside of the tumor. We chose to overcome this by only taking into account the detections in the tumor area, which is how the model is currently used in daily clinical practice [15, 32]. This can be assisted/augmented by including a tumor segmentation model that would indicate beforehand on which part of the WSI to apply the model.

In a limited sample subset we saw that pathologist interaction with the output of the AI model did improve correlation and agreement with the original light‐microscopic MC as has been shown by similar studies [16], so we strongly feel that, in daily diagnostic practice, the output of the algorithm must be presented in a way that allows easy review by the pathologist. That means that the result will only be accepted after a specialist has reviewed all objects detected and clicked on ‘submit’ to detect the 2 mm^2^ area with the most confirmed mitoses. In our implementation, we choose to show both the objects above the optimal *F*_1_‐score detection threshold which are labeled as ‘mitosis’ in the output as well as the objects that are above the optimal *F*_2_‐score threshold which are labeled as ‘negative’. The pathologist can then review both what the model has labeled as mitosis as well as objects that were rejected. That helps counter a potential confirmation bias while speeding up the mitosis review process. Objects can be corrected by dragging thumbnails from one class to the other, or by keyboard correction (key 1 for mitoses, key 2 for nonmitoses) after clicking the thumbnails and inspecting the objects at high resolution. The algorithm runs fully automatically in the background on all our breast slides and, within the PACS, the results can with one click be pulled up for display for each individual image that contains cancer according to the observer. The strong points of this study include the large cohort with long‐term prognostic value, the utilization of both uni‐ and multivariate survival analysis, and the integration of our AI pipeline, consisting of the mitosis detection model and automatic area selection method, in the Sectra PACS. Further studies will address multiobserver reproducibility and the economic impact for a laboratory through time saving of this tedious task.

In conclusion, introducing an AI algorithm in clinical practice comes with several challenges that go above and beyond designing and training a model to perform on fixed datasets. In this study, our open source, publicly available fully automated MC AI algorithm was validated in a large cohort of BC with regard to retained prognostic value compared with traditional light‐microscopic MC.

## Author contributions statement

NS, MA and MV conceived and carried out experiments and analyzed data. FW developed the model used for the analysis. SdV developed the method for the automatic area analysis. All authors were involved in writing the paper and had final approval of the submitted and published versions.

## Supporting information


**Supplementary materials and methods.** Automatic area selection algorithms
**Figure S1.** Examples of the bounded area maximum enclosing convex hull applied on the same tumor area for a different set of detections

## Data Availability

The data that support the findings of this study are available on request from the corresponding author, NS. The data are not publicly available due to identifying information present in the dataset.
