# Supplementary material for: Breast cancer survival prediction using an automated mitosis detection pipeline
Source: J Pathol Clin Res. 2024 Oct 28;10(6):e70008. doi: 10.1002/2056-4538.70008 (PMC11514500; doi:10.1002/2056-4538.70008)
Supplement: Supplementary file 1 — Supplementary materials and methods. Automatic area selection algorithms Figure S1. Examples of the bounded area maximum enclosing convex hull applied on the same tumor area for a different set of detections [file CJP2-10-e70008-s001.pdf]

# Breast cancer survival prediction using an automated mitosis detection pipeline

N Stathonikos *et al.* *J Pathol Clin Res* https://doi.org/10.1002/2056-4538.70008

## Supplementary materials and methods
## Supplementary Figure S1

## Supplementary materials and methods

## Automatic area selection algorithms

### General description

Calculating an area of a fixed size using a set of points gave rise to the following problem from the field of computational geometry: We are given set of points $P$ in the Euclidean plane. The objective is to find within these points a subset $S \subset P$ containing a maximum number of these points such that the Convex Hull (CH) of $S$, that can be defined by the area between the points $S_{CH} \subset S$, has an area equal to at most 10 High Power Fields, or equivalently 2 mm$^2$. We phrase this as an optimization problem for finding a convex area $A_{CH}$ defined by the line segments between points in $S_{CH}$ such that $|S|$ is as large as possible, while the following algorithmic constraints are fulfilled: (1) $S_{CH}$ forms the Convex Hull of $S$ and (2) $A_{CH} \leq 2$ mm$^2$. This problem, which we name the Bounded Area Maximum Enclosing Convex Hull problem, has not been widely studied.

**Exact algorithm**

The exact BAME CH algorithm combines the Gift Wrapping (Jarvis March [27]) strategy with Depth First Search (a divide and conquer approach) and its pseudocode can be seen below - Exact BAME Convex Hull. The algorithm takes all of the detections of the AI model as the points $p_i \in P$ contained in a patch and iterates over these, computing for each the set of points $S_{CH}$ that contains $p_i$ that defines the a $CH$ such that the area constraint is fulfilled and $S$ contains the maximum number of points. This is achieved by iteratively constructing a path from $p_i$ to every other point $p_j$ where $j \neq i$, in the order of the sorted angles these paths make with respect to the x-axis. For each path, all points with a smaller path angle then that of the current path are discarded and the algorithm is then recursively applied with $p_j$ as the new starting point and all points with a larger path angle as remaining points. During each iteration, a check if performed to verify that a path back from $p_j$ to the starting point $p_i$ results in an area that satisfies the area constraint. As such, when all possible options have been explored the area with the highest MC is returned for each $p_i$. Finally, we iterate over all points and return the convex polygon corresponding to the $p_i$ for which the area with the highest MC as the result for the patch.

## **Algorithm:** Exact BAME Convex Hull

Input: $n$ points $p_1,...,p_n \in P$ in the plane, maximum area $A$.

Output: mitotic count, size of area, points $p_i \in S_{CH}$ that define a Convex Hull of $area \leq A$, enclosing as many points from $P$ as possible.

```
function EXACT-BAME-CH(P, A)
    n ← |P|
    if n < 3 then   ▷ ill-defined, as a line does not have an area
        return None
    for i = 1,...,n do
        path ← p_i
        P′ ← P \ p_i          ▷ the remaining points
```

```
        interior ← ∅
        result[i] ← CH-Recursion(path, P', A, interior)
    return argMax_i(result[i].mitosis)    ▷ the CH containing the most points

function CH-RECURSION(path, P', A, interior)
    best-result ← None
    path_pot ← path + path.start    ▷ the potential path
    θ ← angle-wrt-x-axis(path.end, path.start)
    if |path| > 2 then
        area_pot ← polygon-area(path_pot)    ▷ the potential area
        if area_pot > A then
            return None
        if |P'| = 1 then
            point ← P'.start
            θ_point ← angle-wrt-x-axis(path.end, point)
            P' ← ∅
            If point-in-interior(θ, θ_point) then
                interior.append(point)
            else
                path_new ← path + point
                best-result ← CH-Recursion(path_new, P', A, interior)
        else if |P'| > 1 then
            P_sorted' ← sort-angles(path.end, P')
            P_collinear' ← find-collinear(P_sorted')    ▷ for each point save collinear points closer to path.end
            for all point ∈ P_sorted' do
                interior_temp ← interior + Pcollinear' (point)
                if point-in-interior(θ, point.angle) then
                    P'.remove(point)
                    interior.append(point)
                else
                    P' ← eliminate-by-angle(point, P_sorted')    ▷ efficient elimination due to sorted angles
                    path_new ← path + point
                    result ← CH-Recursion(path_new, P', A, interior_temp)
                    if result != None and result.area > 0 then
                        if best-result = None or result.mitosis > best-result.mitosis then
                            best-result ← result
    if best-result = None and |path| > 2 then
        mitosis_pot ← |path_pot| + |interior|
        best-result ← mitosis_pot,area_pot,path_pot
    return best-result
```

**Heuristic BAME CH algorithm**

The heuristic BAME CH algorithm, combines a greedy step in which the convex hull is shrunk with memorization to increase its efficiency. Its pseudocode is presented below - Heuristic BAME Convex Hull. The algorithm starts by constructing the CH defined by $S_{CH}$ for the given points $p_i \in P$ within a patch. Thereby the first constraint, that of the convexity of the solution, is immediately satisfied. In all likeliness, this CH will have an area larger than permitted by the second constraint, however. The algorithm therefore repeatedly shrinks the original CH using a greedy approach, while ensuring that after each operation the solution remains a convex polygon. The shrinking step consists of looking at the triangle between three subsequent points on the current $S_{CH}$. The area and number of points lost that result from reducing this triangle by either changing the middle point to any other point within the triangle, or by discarding the triangle as a whole are computed and saved for future shrinking steps. After having iterated all subsequent sets of three points in $S_{CH}$ , the CH is adjusted such that the area lost per point lost is the greatest. This step is repeated until the total area satisfies the second constraint.

**Algorithm:** Heuristic BAME Convex Hull

Input: n points p1, ..., pn ∈ P in the plane, maximum area A.

Output: mitotic count, size of area, points pi ∈ SCH that define a Convex Hull of area ≤ A, aiming to enclose as many points from P as possible.

```
function HEURISTIC-BAME-CH(P, A)
    n ← |P |
    if n < 3 then   ▷ ill-defined, as a line does not have an area
        return None
    else
        CH ← convex-hull(P )
        idx_rem ← indices(P )  ▷ the indices of the remaining points
        idx_CH ← sort-from-bottom(CH)  ▷ necessary for the CH-Shrinkage step
        drop_dict ← Ø
```

```
        A_CH ← calculate-area(CH)
        while A_CH > A and |idx_rem| > 3 do
            idx_rem, idx_CH , drop_dict, A_removed ←
                CH-Shrinkage(P , idx_rem, idx_CH , drop_dict, A_CH , A)
            A_CH ← A_CH − A_removed
    return |idx_rem|, A_CH , P [idx_CH ]

function CH-SHRINKAGE(P , idx_rem, idx_CH , drop_dict, A_CH , A)
    for all idx ∈ |idx_CH | do
        idx_start ← idx_CH [idx] ▷ get indices of subsequent points on the CH
        idx_middle ← idx_CH [idx + 1 % |idx_CH |]
        idx_end ← idx_CH [idx + 2 % |idx_CH |]
        point_start, point_middle, point_end ←
            P [idx_start], P [idx_middle], P [idx_end]
        A_triangle ← triangle-area(point_start, point_middle, point_end) ▷ Area that can be saved
        if (idx_start, idx_middle, idx_end) /∈ drop_dict then ▷ triangle has not been analyzed previously
            θ_first ← angle-wrt-x-axis(point_start, point_middle)
            θ_second ← angle-wrt-x-axis(point_start, point_end)
            idx_eligible ← ∅
            for all idx_inner ∈ idx_rem \ idx_CH do
                θ_point ← angle-wrt-x-axis(point_start, P [idx_inner ])
                if angle-inside(θ_first, θ_second, θ_point) then
                    idx_eligible.append(idx_inner)
            args ← P, idx_start, idx_middle, idx_end, idx_eligible, A_triangle
            drop_dict ← shrink-step(args) ▷ iterate over the eligible points to calculate the area lost per point dropped, return maximum
            changekey ←find-key-to-adjust(drop_dict)
            idx_CH , idx_rem, A_lost ←adjust-CH(changekey) ▷ adjust the convex hull such that the area lost per point dropped is the greatest
    return idx_CH , idx_rem, drop_dict, A_lost
```

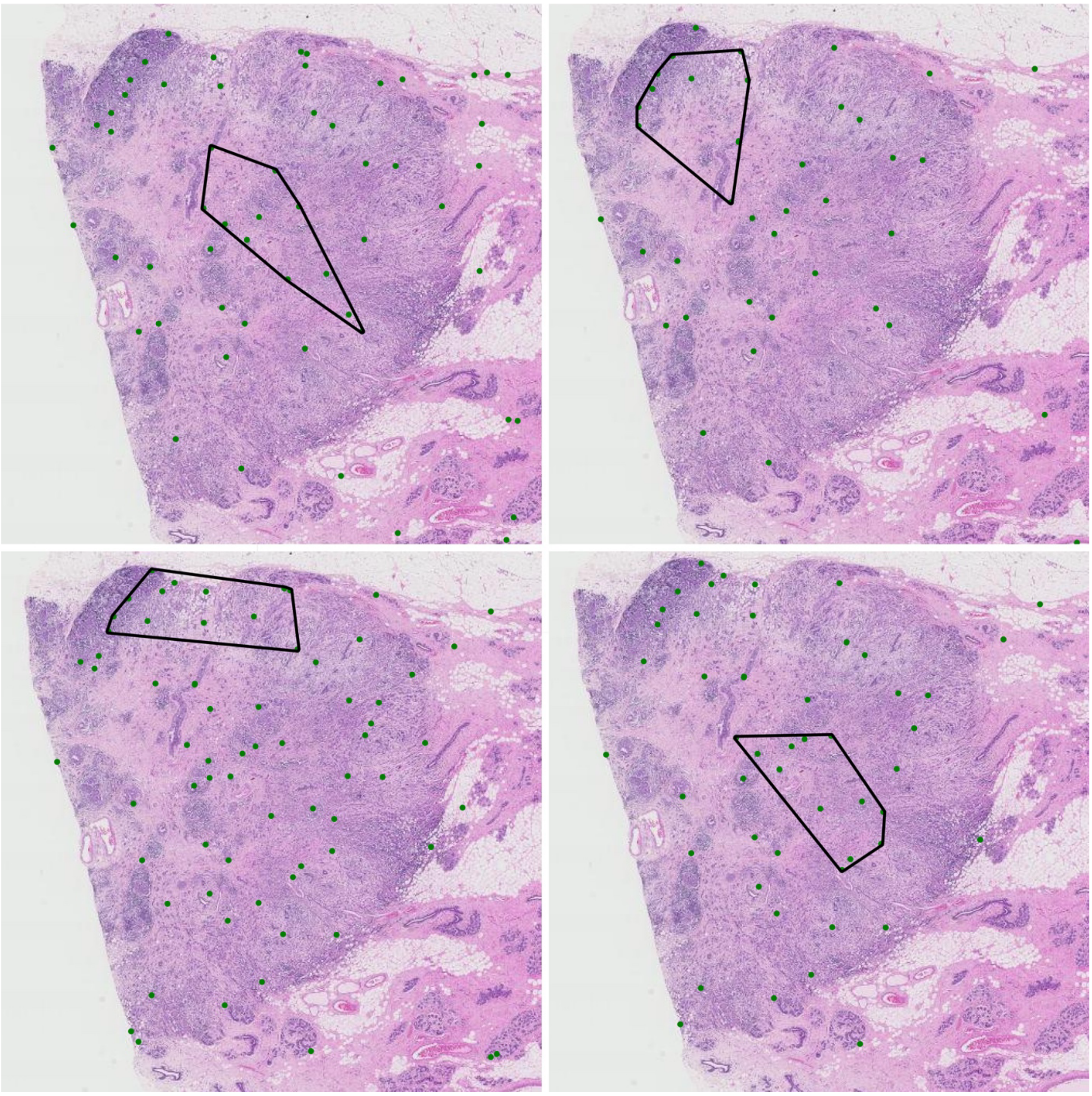

**Figure S1.** Examples of the bounded area maximum enclosing convex hull applied on the same tumor area for a different set of detections. The algorithm will always to try to maximize the amount of mitotic figures for a specific area.
